# The Friend of Clergymen

**Published:** 1871-09

**Authors:** 


### THE FRIEND OF CLERGYMEN.

In the Ohio River, on a little island, Gibraltar by name, is built a commodious country-house, with ample grounds around, with extra accommodations for twelve invited guests, the time of whose stay is designated in the letter of invitation, which letters are sent to clergymen only, without distinction of sect, the only requisite being that they are worthy men, worthy of their calling, and working in it, and who have not the means of "going abroad," or of spending the summer at the costly hotels of the seaside and the Spa. The generous host is the great financier of the age, who has made his millions, and chooses thus to spend a portion of it every year. Here, for a fortnight, these favored clergymen forget the wear and tear and worry and hard work of a faithful minister's life, and in bountiful and beauteous surroundings pass their blest vacations, and then make way for others.

This is a proper appreciation of clergymen, and it is to be hoped that the time is not far distant when they will be more nearly estimated at their proper worth and value, for they are "good men;" and "whatsoever ye do unto them ye do unto me," will be written in the Lamb's Book of Life opposite the names of all such good doers as Jay Cooke and the men who "go and do likewise" in this and kindred ways, with a motive which sanctifies.

